# Anti-Tumor Activity of Novel Nimotuzumab-Functionalized Gold Nanoparticles as a Potential Immunotherapeutic Agent against Skin and Lung Cancers

**DOI:** 10.3390/jfb14080407

**Published:** 2023-08-01

**Authors:** Mohammad Anisuzzman, Varsha Komalla, Mariam Abdulaziz M. Tarkistani, Veysel Kayser

**Affiliations:** Sydney Pharmacy School, Faculty of Medicine and Health, The University of Sydney, Camperdown, NSW 2006, Australia

**Keywords:** nimotuzumab, gold nanoparticle, nanoconjugates, target drug delivery, cellular uptake, cell viability

## Abstract

The epidermal growth factor receptor (EGFR) is vital for many different types of cancer. Nimotuzumab (NmAb), an anti-EGFR monoclonal antibody (mAb), is used against some of EGFR-overexpressed cancers in various countries. It targets malignant cells and is internalized via receptor-mediated endocytosis. We hypothesized that mAb-nanoparticle conjugation would provide an enhanced therapeutic efficacy, and hence we conjugated NmAb with 27 nm spherical gold nanoparticles (AuNPs) to form AuNP-NmAb nanoconjugates. Using biophysical and spectroscopic methods, including ultraviolet-visible spectroscopy (UV-Vis), transmission electron microscopy (TEM), dynamic light scattering (DLS), nanoparticle tracking analysis (NTA), sodium dodecyl sulfate-polyacrylamide gel electrophoresis (SDS-PAGE), and Fourier-transform infrared spectroscopy (FTIR), the AuNP-NmAb complex was characterized. Furthermore, in vitro studies were performed using a medium-level EGFR-expressing skin cancer cell (A431, EGFR^medium^) and low-level EGFR-expressing lung cancer cell (A549, EGFR^low^) to evaluate anti-tumor and cellular uptake efficiency via MTT assay and single-particle inductively coupled plasma mass spectrometry (spICP-MS), respectively. In comparison to NmAb monotherapy, the AuNP-NmAb treatment drastically reduced cancer cell survivability: for A431 cells, the IC_50_ value of AuNP-NmAb conjugate was 142.7 µg/mL, while the IC_50_ value of free NmAb was 561.3 µg/mL. For A549 cells, the IC_50_ value of the AuNP-NmAb conjugate was 163.6 µg/mL, while the IC_50_ value of free NmAb was 1,082.0 µg/mL. Therefore, this study highlights the unique therapeutic potential of AuNP-NmAb in EGFR+ cancers and shows the potential to develop other mAb nanoparticle complexes for a superior therapeutic efficacy.

## 1. Introduction

The epidermal growth factor receptor (EGFR) is a transmembrane-bound glycoprotein that regulates cell signaling in the human body. EGFR is often highly upregulated in cancer cells where it induces multiple signaling cascades that stimulate cell proliferation and angiogenesis, which lead to pathogenesis and the progression of cancer [1,2]. Thus, a high EGFR expression is associated with declined clinical outcomes in several epithelial cancers, such as head and neck cancer and breast cancer [2,3,4,5]. Therefore, targeting aberrant EGFR represents an appealing strategy for EGFR-expressing cancer treatment interventions.

Monoclonal antibodies (mAbs) are among the most effective anti-EGFR medicines due to being highly specific for target receptors and enjoy major success in the clinical setting. They can also engage with the immune system against cancer, are serum-stable, and have a long serum half-life [5]. Currently, there are four marketed EGFR mAbs (cetuximab, necitumumab, NmAb, and panitumumab), approved as therapies for some epithelial cancers and more than a dozen of new mAbs are undergoing clinical trials [6,7]. NmAb is a humanized IgG1 antibody and interacts with the domain III of the extracellular amino acid (R353, S356, F357, T358, and H359T) of EGFR, thereby inhibiting the downstream EGFR signaling cascade [7]. Apart from blocking EGFR signaling, it also abrogates tumor growth by activating natural killer cells via antibody-dependent cellular cytotoxicity (ADCC), and also induces adaptive immunity via tumor antigen-specific T cells [8]. Currently, NmAb is approved in Argentina, Colombia, Cuba, Gabon, India, Ivory Coast, Peru, Sri Lanka, and Ukraine for treating head–neck squamous cell carcinoma; in Argentina, Cuba, Philippines, and Ukraine for glioma (adult and pediatric); and in China for nasopharyngeal cancer [9]. NmAb has an orphan drug status in the United States and the European Union for pancreatic cancer and diffuse intrinsic glioma [10].

Even though mAbs are highly specific for malignant cells, they have certain limitations. For instance, cetuximab and trastuzumab have limited distribution and target-binding capability within solid tumors in mice and, hence, higher doses are necessary to achieve a uniform distribution throughout the tumor [11]. Recent studies have shown that mAb-modified nanoparticles have the potential to target tumor cells selectively, improve internalization, and demonstrate superior anti-proliferative efficacy compared to that of mAb itself [12,13]. In addition, nanoparticle-based drug delivery systems can preferentially amass in tumors by increased penetration and retaining the boosted permeability and retention (EPR) effect. Coupling nanoparticles to mAbs might allow combining effective “passive” delivery with an active targeting ability, which would be specifically advantageous in the treatment of solid tumors comprising heightened levels of angiogenesis and leaky vasculature [14].

In this work, we conjugate NmAb onto AuNPs by PEGylated thiol conjugation via amide bonds. AuNPs are chemically inert with minimal toxicity and generally do not have immunogenicity [15,16]. Polyethylene glycol (PEG) is frequently applied to stabilize AuNPs. The amphiphilic nature of PEG makes AuNPs dispersible within an aqueous environment and ensures their stability in biological media [17]. Moreover, PEGylated AuNPs with a 100–200 nm size are also “stealth” nanoparticles since they can escape detection by the immunological system and have a long serum half-life [18]. AuNPs are attractive candidates for the conjugation of antibodies and other proteins due to their ease of production and functionalization [19].

We further investigated the direct comparison of cellular internalization and cytotoxic effects of NmAb-functionalized AuNPs and NmAb themselves with different levels of EGFR-expressing cancer cells for skin (A431) and lung (A549). Our results indicate that AuNP-NmAb showed improved anti-tumor activity and cellular uptake towards EGFR-overexpressing cancer cells over free NmAb. To our knowledge, this is the first study to report AuNP-NmAb development and characterization and to use in vitro studies using skin and lung cancer cells. This study demonstrates that AuNP-NmAb has a great potential for the treatment of EGFR+ cancers. Our approach could be adapted to develop biobetters with existing monoclonal antibodies.

## 2. Materials and Methods

### 2.1. Materials

The humanized anti-EGFR mAb NmAb, BIOMAb EGFR^®^, was purchased from Biocon Limited (Bengaluru, India). A 5 kDa Thiol-PEG-NHS (NHS-PEG-SH) linker was acquired from Nanocs (Boston, MA, USA). Gold (III) chloride trihydrate, Cis-diammineplatinum (II) dichloride, potassium carbonate, and tannic acid were bought from Sigma-Aldrich (Castle Hill, NSW, Australia). Formvar/carbon-coated copper grids (200 square mesh) were bought from ProSciTech (Kirwan, QLD, Australia). Sodium citrate trisodium salt dihydrate was acquired from Astral Scientific (Taren Point, NSW, Australia). Precision plus protein^TM^ dual-colour standard was purchased from BIO-RAD (Hercules, CA, USA). A431 and A549 cell lines were generous donations of Grewal and Chrzanowski labs, School of Pharmacy, The University of Sydney, Australia. Thiazolyl blue tetrazolium bromide, trypsin/EDTA solution (TE), fetal bovine serum, and penicillin and streptomycin antibiotic (10,000 U/mL and 10,000 µg/mL) were purchased from ThermoFisher Scientific (Macquarie Park, NSW, Australia). Low-glucose-containing Dulbecco’s modified Eagle medium (DMEM) and high-glucose-containing Dulbecco’s modified Eagle medium (DMEM) were bought from Life Technologies (Mulgrave, VIC, Australia). Trypsin/EDTA solution (TE) and phosphate-buffered saline (PBS) tablets were purchased from Sigma-Aldrich (Macquarie Park, NSW, Australia). Millex-GV syringe filter (0.22 µm, PVDF) and all other chemical reagents were obtained from Sigma-Aldrich (Castle Hill, NSW, Australia) and directly used without additional purification. In-house MilliQ (18 Ωcm^−2^) filtration system (Millipore) water was used for all the experiments.

### 2.2. Methods

#### 2.2.1. Synthesis of Spherical Citrate–Tannate-Capped AuNPs

The synthesis of a 27 nm colloidal monodispersed AuNP solution was performed by using complex reducing agents. Briefly, 150 µL of 22 mM trisodium citrate (TSC), 50 µL of 2.5 mM tannic acid (TA), and 50 µL of 150 mM potassium carbonate (K_2_CO_3_) were poured into a conical flask containing 30 mL of Milli-Q water and vigorously stirred at 60 °C for around 5 min. After that, 1.25 mL of 12.5 mM HAuCL_4_ solution was gradually added to the previous mixture and vigorously stirred for an extra 2 min at 60 °C. The temperature was then further reduced to 40 °C as soon as the mixture’s color changed from pale yellow to blackish, and it was stirred gently for another 15 min. Finally, AuNP synthesis was verified by the color of the reaction solution from deep violet to wine-red color. The colloidal solution of AuNPs was allowed to cool. Following cooling, the optical density (O.D._531_) of the produced solution was corrected. (O.D._531_) to 1.0 with the Milli-Q water to obtain 27 nm PEGylated bare AuNPs and finally stored at 4 °C.

#### 2.2.2. PEGylation of NmAb and AuNPs and Surface Functionalization of AuNPs (AuNP-NmAb)

NmAb antibody concentration was determined using the extinction coefficient *Ɛ*_280_ = 2.25 × 10^5^ M^−1^ cm^−1^ and diluted to 5 mg/mL. Before the conjugation, a Thiol-PEG-NHS linker (5 kDa) stock solution (20 mg/mL, 4 mM) was prepared freshly each time in NaHCO_3_ 0.01 M pH 8 and used immediately as NHS ester suffers from hydrolysis at this pH. A total of 20 µL NmAb (5 mg/mL) and 10 µL Thiol-PEG-NHS linker stock solutions were combined in a small tube and incubated for 12 h night in the 4 °C room under moderate rotation for PEGylation of NmAb. AuNPs were centrifugated at 5000 rpm and designed for 10 min to obtain AuNP pellets, while the temperature was maintained at 15 °C. The Thiol-PEG-NmAb complex was added up to the AuNP pellets and incubated overnight under continuous stirring at 4 °C for enabling the proper replacement of citrate–tannate ion by the thiol group. The unreacted Thiol-PEG-NHS linker covered the empty surface of the unconjugated AuNPs to ensure a stable AuNP solution. Finally, covalently conjugated AuNP-NmAb were resuspended to a suitable volume with an O.D._531_ value of 1.0 using NaHCO_3_ 0.1 M pH 8.0 buffer [12]. For the PEGylation of bare AuNPs, the same procedure was followed using only thiol-PEG. NmAb-PEG-SH was obtained by incubating with 60 times excess of the Thiol-PEG-NHS linker’s molar concentration when compared to free NmAb. Meanwhile, AuNP-NmAb was obtained by using 2280 times excess of NmAb’s molar concentration and 133,333 times excess of Thiol-PEG-NHS linker’s molar concentration when compared to AuNPs.

#### 2.2.3. Characterization of Nanoparticles

##### Transmission Electron Microscope (TEM)

The core diameter, particle aggregation, and morphology of AuNPs before and after conjugating to NmAb were examined using TEM (JEOL JEM 1400, Tokyo, Japan) in the Australian Centre for Microscopy & Microanalysis (ACMM) at The University of Sydney. Briefly, 8 µL of diluted AuNP aqueous solution (dilution ratio 1:3) were dropped onto the copper grid and air-dried overnight. The TEM was run at a 120 kV accelerated voltage. The TEM pictures were processed using ImageJ-win64 software (version 1.53g).

##### Dynamic Light Scattering (DLS)

The mean hydrodynamic diameter (Z-average size), polydispersity index, and zeta-potential of AuNPs were measured using a Zetasizer Nano ZS (Malvern Instruments Ltd., Malvern, UK). Using a 633 nm helium–neon laser, scattered data were collected at an angle of 173° to incident light at 25 °C. The nanoparticle charges were determined using the same instrument for the zeta potential measurements. The samples were diluted (1:2 for DLS and 1:2000 for zeta potential) with the filtered (0.2 µm) Milli-Q water. Individual particle Brownian motion was transformed into particle size, which was determined using the Stokes–Einstein equation.

##### Nanoparticle Tracking Analysis (NTA)

The hydrodynamic diameters of the bare and NmAb-conjugated AuNPs were measured using a NanoSight NS300 (Malvern Panalytical, Malvern, UK) featuring a top-tier sCMOS camera and a 405 nm laser module. The sample chamber was rinsed thoroughly with filtered MilliQ water and ethanol to remove any particles and air bubbles before the start of the measurements. Using a disposable syringe, around 500 µL of the sample (PEGylated bare AuNPs or AuNP-NmAb) was placed in the chamber. Three videos of the particle movement, i.e., Brownian motion, of 1 min each, were recorded for each sample. For all the measurements, filtered Milli-Q water was used as a dispersant. Data analyses were performed using the instrument software (NanoSight™) v 3.4.

##### Fourier-Transform Infrared Spectrometer (FTIR)

Infrared spectra of PEGylated bare AuNPs or AuNP-NmAb were obtained using a PerkinElmer spectrum 100 Fourier-Transform Infrared Spectrometer (PerkinElmer, Waltham, MA, USA). Briefly, a volume of 10 µL of the sample (PEGylated bare AuNPs or AuNP-NmAb) was applied to the attenuated total reflectance (ATR) crystal and dried at room temperature for 1 h until a thin coating remained. Isopropyl alcohol (IPA) was used as a blank for each sample. All spectra were recorded in wavenumbers ranging from 600 to 4000 cm^−1^ from 120 scans on average.

##### Sodium Dodecyl Sulphate Polyacrylamide Gel Electrophoresis (SDS-PAGE)

SDS-PAGE was employed to validate the conjugation of NmAb with AuNPs. Protein or AuNPs were initially mixed with loading buffer 1× (deionized water/glycerol (4:3, *v*/*v*), 1 M tris-HCL, 20% (*w*/*v*) SDS, 0.5% (*w*/*v*) bromophenol blue, and 0.7 M b-mercaptoethanol) and boiled for 10 min at 100 °C. Each sample was placed on a 15% polyacrylamide with a volume of 10 µL. To accomplish gel separation, samples were electrophoresed for 1.5 h at 125 V using tris–glycine–SDS running buffer. Subsequently, the protein concentrations were assessed by treating the gels in a Coomassie brilliant blue R-250 coloring solution and then soaking them in a de-staining solution (20% methanol and 10% glacial acetic acid (*v*/*v*)). SDS is an ionic surfactant that denatures proteins and binds to them, making them uniformly, negatively charged and allowing them to move over the gel to the positively charged electrode. Finally, the casting gels were then transferred into distilled water, and the corresponding images were acquired using the Bio-Rad Gel Doc™ XR system with Bio-Rad Image Lab™ Software (Bio-Rad Laboratories Pty., Ltd., Hercules, CA, USA). A 10–250 kDa Precision plus protein^TM^ dual-color marker was used as molecular mass standard in the SDS-PAGE measurements.

##### UV-Vis Spectroscopy

The UV-Vis absorption spectrum of PEGylated bare AuNPs and AuNP-NmAb were measured in a wavelength range between 200 nm and 800 nm with a 0.5 nm resolution via a Shimadzu 2600 UV-Vis spectrophotometer (Shimadzu, Japan). Blank subtraction with a medium (DMEM) or distilled water was performed before recording the measurements for samples in the corresponding solutions, respectively, as well as the absorbance. The absorbance of AuNPs is proportional to the nanoparticle concentration, according to the Beer–Lambert law.

(1) $$\mathrm{Beer}–\mathrm{Lambert}\;\mathrm{equation},\;A=log\left(\frac{l_{0}}{I}\right)=\varepsilon .c.l.A$$

where A, I_0_, I, c, l, and ε are the absorbance, incident light intensity upon the specimen cell, the intensity of light leaving the specimen cell, molar concentration, optical path length, and molar extinction coefficient, respectively.

##### Colloidal Stability in Salt and a Biological Medium by UV-Vis and DLS

In vitro stability studies were performed on PEGylated bare AuNPs or AuNP-NmAb conjugates [20,21]. Briefly, the aqueous solution of 10% NaCl and 10% fetal bovine serum (FBS)-containing media (DMEM) was mixed with either PEGylated bare AuNPs or AuNP-NmAb nanoconjugates in a ratio of 1:4 *v*/*v*. Color shifts were used to assess the stability of the nanoconjugates. UV absorbance, DLS, and zeta potential measurements were conducted after 24 h.

##### Calculation of Antibody Aggregation and Antibody-Binding Percentage through UV-Vis Spectroscopy

The UV-Vis absorbance peaks of bare AuNP-Thiol-PEG and AuNP-NmAb were at 532 nm and 536 nm, respectively. The UV-Vis absorbance peak red-shifted to 650 nm due to aggregation. The aggregation parameter (AP) was calculated by the following equation:

(2) $$AP=\frac{A650nm-A_{ref}650nm}{A532nm-A_{ref}532nm}$$

where A_ref_650 nm and A_ref_532 nm were the absorbances of water at 650 nm and 532 nm, respectively, and A650 nm and A532 nm were the absorbances of PEGylated bare AuNPs at those wavelengths.

Any number below 1.0 indicates that there is no particle aggregation, i.e., a stable suspension. A similar equation was used for the conjugated AuNPs to calculate the aggregation parameter but replaced by a UV-Vis absorbance value of 536 nm instead of a 532 nm UV-Vis absorbance value [22]. Particle aggregation was defined as a computed value greater than 1.0 [23]. More UV-Vis measurements were conducted to see how much unbound antibody was still in the supernatant. After that, the antibody-binding efficiency was determined using the following equation:

(3) $$Binding\;efficiency\%=\frac{\left[a{Ab}_{0}\right]-\left[aAb\right]}{\left[a{Ab}_{0}\right]}\times 100$$

where aAb_0_ is the absorbance of the AuNP-NmAb conjugate solution at λ_max_ 280 nm and aAb is the absorbance of the washed supernatant at λ_max_ 280 nm.

#### 2.2.4. In Vitro Studies

##### Culture of A431 and A549 Cells

A431 is an immortalized epidermal (skin) carcinoma cell line and was maintained in low-glucose DMEM, while A549 is a lung carcinoma epithelial cell line and was maintained in high-glucose DMEM. Both media were supplemented with fetal bovine serum (FBS) at 10% (*v*/*v*) and penicillin/streptomycin at 1% (*v*/*v*). Every two days, the media were replaced.

##### Determination of the Percentage of Cell Viability through MTT Assay

The viability percentage of A431 and A549 cells following exposure to AuNPs and Au-NmAb was calculated via MTT (3-(4,5-dimethylthiazol-2-yl)-2,5-diphenyltetrazolium bromide) assay. A431 or A549 cell lines (4 × 10^3^ cells/well) were deposited in 96-well flat bottom well plates containing 10% FBS and subjected to a reaction for 24 h at 37 °C. Cisplatin, a strong anti-cancer drug, was chosen as the standard control. The medium was then removed, and the cells were treated with cisplatin, NmAb, Au-Thiol-PEG, or AuNP-NmAb at varying doses for 72 h in fresh media without FBS. After that, the cells were rinsed with PBS. A total of 20 μL (5 mg/mL) of the MTT solution and 80 μL of fresh medium were added into each well. Following 3 h incubation at 37 °C, the medium was discarded and 100 μL of cell-culture-grade DMSO was added into each well in order to dissolve the live cells and produce MTT-formazan crystals (blue—relative to the number of living cells) in the presence of mitochondrial enzymes. Each well’s absorbance value was determined using a PerkinElmer Victor X4 multilabel plate reader (PerkinElmer, Inc., Waltham, MA, USA) at 570 nm. Further, cytotoxicity was measured as a percentage of the cell viability. The untreated cells were considered 100% viable and the empty wells containing DMSO were chosen for background correction [24]. The following equation was used to calculate the cell viability percentage (%):

(4) $$Percent\;cell\;viability\left(\%\right)=\frac{\left[A_{570}(treated\;cells)-background\right]}{\left[A_{570}(controlled\;cells)-background\right]}\times 100$$

where A_570_ is the absorbance at 570 nm. IC_50_ values were calculated according to 4 parameters’ sigmoidal logistic curves plotted on a logarithmic scale using GraphPad Prism 8 software (version 8.4.3).

##### Estimation of Cellular Uptake of AuNPs through ICP-MS Analysis

A 3 × 10^4^ cells/well cells were placed in a 24 well plate of A431 or A549 in 10% FBS-containing medium and incubated for 24 h at 37 °C. Then, both cells were treated with AuNPs or AuNP-NmAb conjugates and left for a further 24 h. Nanoparticle-containing media were eliminated, and cells were purified thrice with 1× PBS pH 7.4 to detach any unbound nanoparticle. The cells were separated from the wells using trypsin-EDTA before being placed in a 1.5 mL low-attaching microcentrifuge tube. Subsequently, trypsin was eliminated through centrifugation at 1000 rpm for 5 min at 4 °C. After that, the cells were washed with 1× PBS pH 7.4 twice and centrifuged again to remove any traces of trypsin. PBS was used to resuspend the cell pellet up to 1.0 mL for counting the total number of cells. The obtained cell pellet was processed in 200 µL of nitric acid (15.9 M HNO_3_) by 12 h incubation at room temperature. Further, 600 µL of hydrochloric acid (12.1 M HCL) was added for solubilizing the AuNP. Finally, the samples were diluted in the MilliQ water (1:10) and the spICP-MS test was conducted using a PerkinElmer Nexion 300× spICP-MS instrument (PerkinElmer, Inc., Waltham, MA, USA) calibrated with 0.1, 1, 10, and 100 parts per billion (ppb) of a gold standard solution.

##### Statistical Analysis

The mean ± SD was used to express (*n* = 3) all the data; ^#^ and * indicate *p* < 0.05, ^##^ and ** indicate *p* < 0.01, ^###^ and *** indicate *p* < 0.001, and ^####^ and **** indicate *p* < 0.0001 when compared with the control. Data were represented via GraphPad Prism (GraphPad software version 8, La Jolla, CA, USA). One-way ANOVA post hoc Fisher’s least significant difference (LSD) was utilized for estimating the significance.

## 3. Results

### 3.1. AuNP Synthesis and Surface Functionalization with NmAb

**Figure 1:**
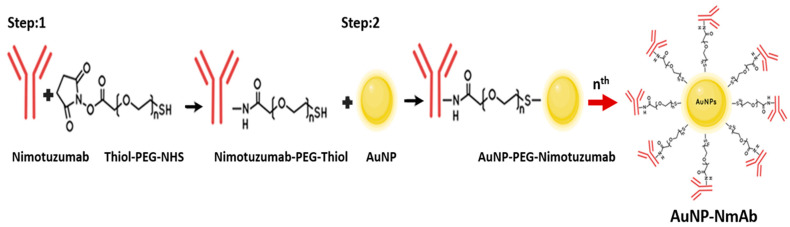
NmAb-functionalized AuNPs. Step 1: NHS ester reaction scheme for chemical conjugation with NmAb (primary amine), and Step 2: AuNP surface functionalization (AuNP-NmAb) using thiol-PEG-NHS NmAb complexes through citrate–tannate ion replacements by thiol group through a cross linking reaction. Not drawn to scale.

**Figure 2:**
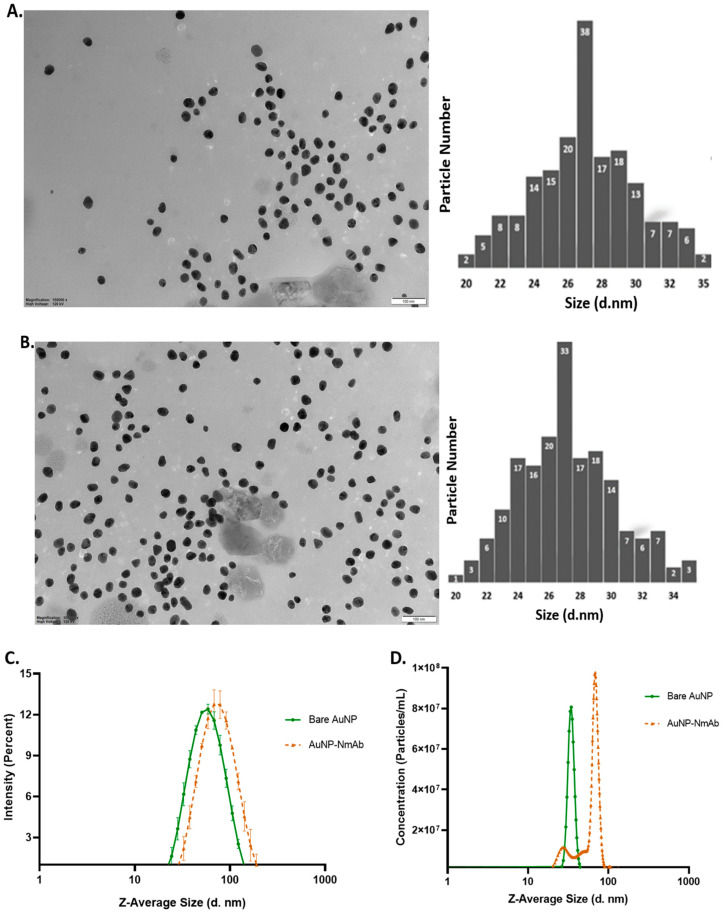
Size, shape, and morphology of AuNPss via transmission electron microscopic (TEM), dynamic light scattering (DLS), and nanoparticle tracking analysis (NTA) diagram. (**A**) TEM image and size histogram of PEGylated bare AuNPs (*n* = 104 particles) using a 100 nm scale bar. (**B**) TEM image and histogram of AuNP-NmAb conjugates (*n* = 180 particles). (**C**) Hydrodynamic diameter of PEGylated bare AuNPs and conjugated AuNP-NmAb nanoparticles as determined by DLS. (**D**) The particle size distribution in colloidal solutions via NTA. AuNP size distribution was obtained by calculating the average diameter (d. nm) ± standard deviation.

The 27 nm (average) of a dark red-colored monodisperse spherical AuNP solution was prepared via HAuCl_4_ reduction using capping agents, such as tannic acid, an electron stabilizing agent, and trisodium citrate complex. For the synthesis of various sizes of 10 nm to 30 nm AuNPs, different amounts of HAuCl_4_ and TA-TSC complex were utilized (Figure 1). The UV-vis absorption peak of AuNP-NmAb shows a well-defined and narrow band spectrum. The AuNPs displayed an absorption peak at 531.33 nm with a 27.00 ± 3 nm mean size. The PEG-coated and NmAb-functionalized AuNP sample had an absorption maximum at 535.17 nm (Table 1 and Figure 2), which confirms the conjugation reaction.

### 3.2. Physicochemical Characterization of PEGylated Bare AuNPs and NmAb-Functionalized AuNPs (AuNP-NmAb)

TEM was performed to find the AuNPs’ core diameter using ImageJ-win64 analysis. A homogeneous spherical shape with a monodispersed size distribution was observed for both the PEGylated bare AuNPs and AuNP-NmAb (Figure 2A,B). The average sizes of PEGylated bare AuNPs and AuNP-NmAb were found to be 27.0 ± 3.0 nm and 27.21 ± 3.08 nm, respectively.

The intensity distributions of 27 nm PEGylated bare AuNPs and 10% NaCl, and with 10% FBS-treated AuNP-NmAb, and AuNP-NmAb were centered at 41.77 ± 1.26, 58.3 ± 0.36, 59.81 ± 1.184, and 73.17 ± 1.144 nm, respectively (Figure 2C). The zeta potential also confirmed the surface charge changes in the prepared PEGylated bare AuNPs and AuNP-NmAb. PEGylated bare AuNPs, AuNP-NmAb, and 10% NaCl and 10% FBS-incubated AuNP-NmAb showed surface charges of −35.53 ± 1.7 mV, −0.048 ± 0.10 mV, −10.2 mV, and −8.54 ± 1.33 mV, respectively. The zeta potential value is displayed in Table 1. The polydispersity index for AuNP-NmAb and PEGylated bare AuNPs were found to be 0.264 and, 0.315, respectively (Table 1), which confirms the particle size distribution as monodisperse.

The hydrodynamic mean and mode size of PEGylated bare AuNPs was 34.3 ± 0.2 nm and 34.7 ± 0.2 nm, respectively, and the mean and mode size of AuNP-NmAb was increased to 64.9 ± 2.9 nm and 71.4 ± 1.9 nm, respectively (Figure 2D and Table 1). Thus, AuNP-NmAb is projected to increase in diameter to 60–70 nm relative to the unconjugated AuNP particles. The results for NTA are in line with the results obtained from DLS, which demonstrated a size increase of ~20 to 30 nm following the conjugation of NmAb with AuNPs. Overall, the results indicate that the AuNPs developed in this study are uniform and monodisperse with an increase in size following the conjugation with NmAb.

### 3.3. Determination of the Surface Functionalization of AuNPs with NmAb and Their Stability Analysis

**Figure 3:**
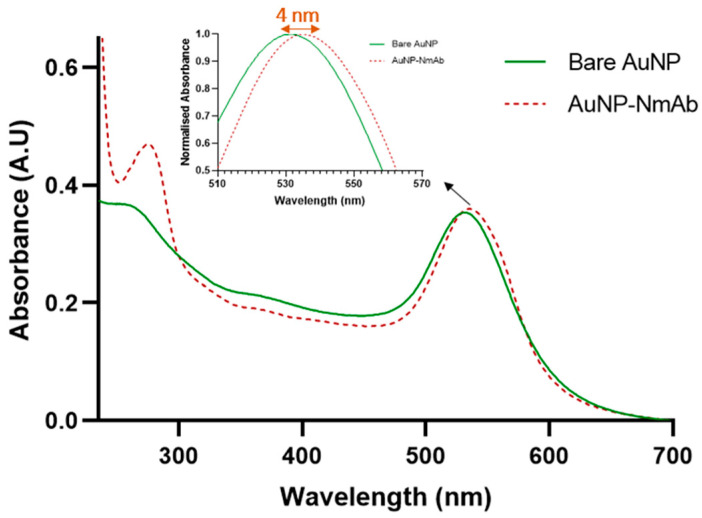
UV-Visible absorption spectroscopy of PEGylated bare AuNPs and conjugated AuNP-NmAb. The inset shows the band displacement caused by the surface modification of AuNPs. The additional peak at the wavelength of 260–280 nm is due to aromatic amino acids in NmAb, indicating the conjugation of NmAb to AuNPs. Data are the average of three measurements.

Following the synthesis of nanoconjugates, the functionalization of AuNPs with NmAb was evaluated using UV-Visible spectroscopy. Figure 3 demonstrates the absorbance of AuNPs with a characteristic surface plasmon resonance (SPR) band at 532 nm absorption maximum (λ_max_). A bathochromic shift of around 4 nm was observed after conjugation with NmAb. The shift indicates the surface functionalization of AuNPs with NmAb. The result λ_max_ was found to be ~27 nm size of AuNPs. In contrast, no absorption peak was observed at the identical wavelength for NmAb and thiol-PEG-NHS linker alone.

The 10% NaCl and 10% FBS cell culture medium-treated PEGylated bare AuNPs were aggregated, while AuNP-NmAb was found to be remarkably stable and did not show any absorbance peak shift. The estimated value of AuNP-NmAb was 0.44, which is lower than 1.0, confirming the stability of the particles in the solution. Around ~92% of NmAb was bound to the AuNP surface via a thiol-PEG-NHS linker, estimated theoretically as mentioned in the experimental section by the Beer–Lambert law using the UV-Vis absorbance peak value (Figure 3 and Table 1). The results indicate no substantial changes, and no precipitates were observed for the conjugated AuNPs in PBS, bicarbonate buffer water, and cell culture medium.

**Figure 4:**
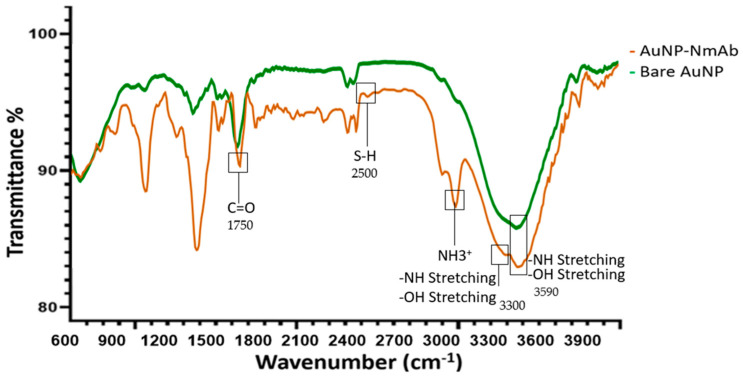
FTIR spectra of AuNP-NmAb (upper trace) and PEGylated bare AuNPs (lower trace) demonstrate functional group changes detected between 600 and 4000 cm^−1^. The bands of COO^−^, –NH, and NH_3_^+^ and the deformation of -SH indicate the NmAb coupling to the AuNP surface. Boxes indicate different functional groups present in the AuNP-NmAb nanoconjugates.

In addition, FT-IR was performed to evaluate the changes in functional groups in AuNPs following bioconjugation to NmAb (Figure 4). Specific peaks of the AuNPs are observed between 1000 and 1650 cm^−1^. Particularly, 3300 cm^−1^ is the region for amide A that was formed due to crosslinking between NmAb and linker NHS, causing a N–H stretch. The band from 1600 to 1700 cm^−1^ indicates the presence of the amide-I band owing to the amide C=O stretching vibrations of the peptide bonds. The characteristic band around 2500 to 2600 cm^−1^ confirms the presence of S-H in the experimental conjugates.

**Figure 5:**
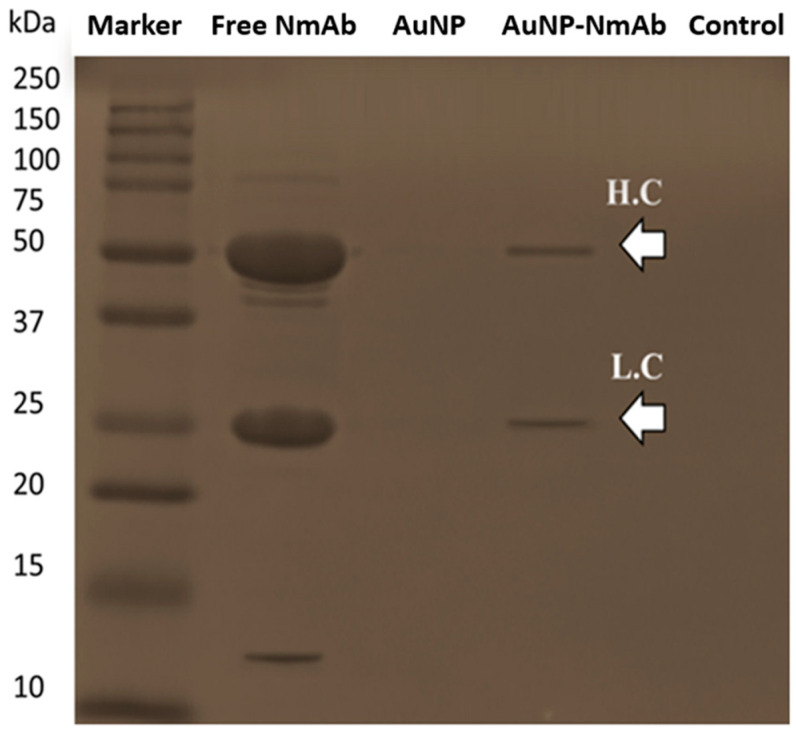
SDS-PAGE gel electrophoresis of the conjugation of NmAb with AuNPs. Equal amounts of AuNPs, AuNP-NmAb, NmAb, the control, and the marker were treated in reducing conditions (heat and β-mercaptoethanol) and loaded onto a 15% polyacrylamide gel. NmAb: free NmAb; AuNP-NmAb: AuNPs conjugated with NmAb; AuNP: AuNPs after PEGylation; M: protein marker (10–250 kDa).

We also studied AuNP-NmAb conjugation via SDS-PAGE (Figure 5). As expected, the AuNPs without functionalization with NmAb produced no protein fragments, whereas the free NmAb showed two characteristic light and heavy chains bands at around 20–30 and 40–60 kDa, respectively. Similarly, the AuNP-NmAb fragments generated a light band at ~50 kDa, which matched the heavy-chain antibody fragment molecular weight. Additionally, there was another band in the 20–30 kDa range, which belongs to the light-chain fragment. The results imply that NmAb was successfully conjugated with AuNPs.

### 3.4. In Vitro Studies

#### 3.4.1. Evaluation of Anti-Tumor Activity in EGFR+ Cancer Cells

**Figure 6:**
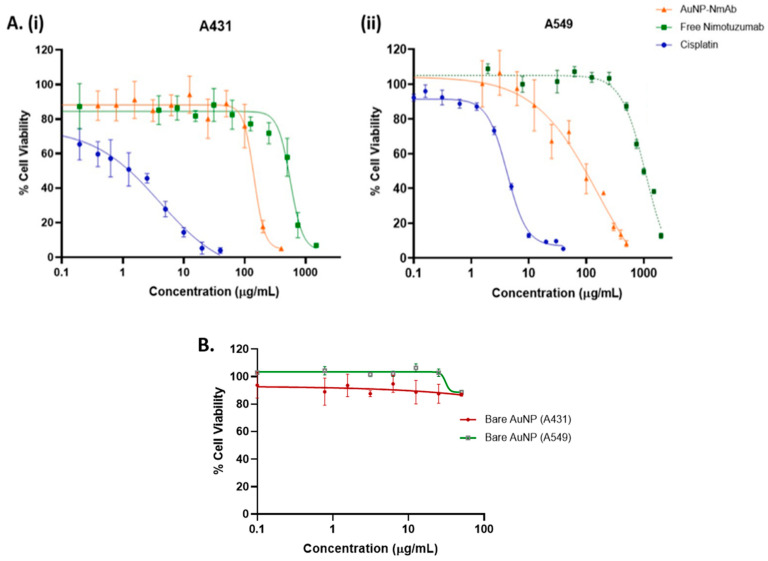
Evaluation of in vitro cytotoxicity and biocompatibility. (**A**) The percentage cell viability as measured by the MTT assay following treatment with AuNP-NmAb, free NmAb, and cisplatin in (**i**) A431 cells and (**ii**) A549 cells. (**B**) The viability % (safe concentration) was determined via MTT assay on A431 and A549 cells after incubation with bare AuNPs. Data are reported as mean ± SD and *n* = 3.

The MTT assay results reveal a dramatic dose-dependent reduction in the % of viable cancer cells for both A431 and A549 cell lines with AuNP-NmAb, NmAb, and cisplatin (Figure 6A,B). AuNP-NmAb has more anti-tumor activity than the free NmAb (~4 to 5-fold) in both cell lines.

AuNP-NmAb and NmAb show significant cytotoxicity with lower IC_50_ values in A431 compared to A549, as indicated in Figure 6A. The IC_50_ values of the AuNP-NmAb conjugate were 142.7 and 163.6 µg/mL, while the IC_50_ values of the free NmAb were 561.3 and 1082.0 µg/mL, for A431 and A549 cells, respectively, around 72 h incubation, attributing the efficacy of AuNP-NmAb to specific EGFR-targeted activity. As expected, cisplatin showed considerably more cytotoxicity against both the cancer cell lines than the free NmAb and AuNP-NmAb with IC_50_ values for A431 and A549 cells at 4.023 and 4.199 µg/mL, respectively (Figure 6A). The obtained results demonstrate a safe profile of AuNPs up to 100 µg/mL concentration for both cells.

#### 3.4.2. Evaluation of the Cellular Uptake of AuNP-NmAb in EGFR+ Cancer Cells

**Figure 7:**
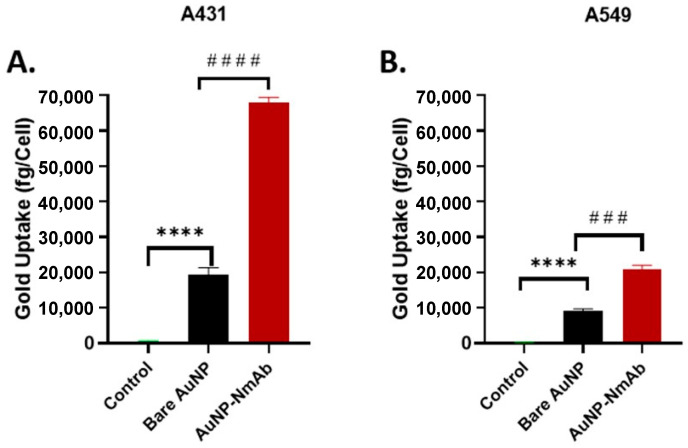
Cellular uptake of the NmAb-functionalized AuNPs into cancer cells detected by ICP-MS. (**A**) A431 and (**B**) A549 cells following 24 h of incubation. AuNP-NmAb vs. PEGylated bare AuNPs ^####^ *p* < 0.0001; PEGylated bare AuNPs vs. Control **** *p* < 0.0001 (A431); PEGylated bare AuNPs vs. Control **** *p* < 0.0001; and AuNP-NmAb vs. PEGylated bare AuNPs ^###^ *p* < 0.001 (A549) (one-way ANOVA and post hoc Fisher’s LSD). Data are presented as mean ± SD and *n* = 3.

The uptake of AuNP-NmAb per cell was considerably superior compared to the equivalent amount of PEGylated bare AuNPs (Figure 7A,B). Although the cellular absorption can be influenced by the surface charge of AuNPs, the presence of NmAb is the main factor for the higher cellular uptake of AuNP-NmAb, which binds to the receptor and enables the internalization of the nanoparticles. On average, 19,387 and 9076 bare AuNPs were able to enter A431 and A549 cells, respectively, while the uptake of AuNP-NmAb was 67,951 and 20,875 by A431 and A549 cells, respectively. Additionally, the zeta potential results demonstrate that AuNP-NmAb has a neutral charge (−0.048 ± 0.10 mV) and AuNPs have a negative charge (−35.53 ± 1.7 mV) (Table 1), indicating the surface functionalization of nanoparticles, which in turn supports the greater internalization of AuNP-NmAb.

## 4. Discussion

The benefits of the targeted delivery of anti-cancer drugs versus the non-targeted administration of therapies are clear. MAbs are preferred cancer therapeutic agents because of their high affinity and specificity for the target. Combining the targeting ability of monoclonal antibody with nanoparticles becomes a viable drug delivery approach [14,25]. In this study, we conjugated AuNPs with NmAb to create advanced EGFR receptor-specific AuNP-NmAb.

For AuNP synthesis, tannic acid and sodium citrate mixture was shown to be a better and strong reducing complex and stabilizing agent compared to tannic acid or sodium citrate alone. In the reduction process, the carboxylic group (COOH) in tannic acid transforms into COO–, which can serve as a surfactant to adhere to the AuNP surface and stabilize it via electrostatic interactions. The mean size of the synthesized AuNP was estimated the UV-Vis spectrum and measured via TEM [26,27,28]. Figure 1 demonstrates the procedure to synthesize NmAb-functionalized AuNPs. PEGylation reinforced the particles to remain consistently dispersed and enhanced the ability to add an increased number of antibodies [20]. The attachment of the NmAb onto the AuNP surface was conducted via a bifunctional NHS-PEG-SH linker (polyethylene glycol linker along with an N-hydroxy succinimide ester end and a thiol group). The NmAb-functionalized AuNPs changed their deep red color to a faintly purple-red color, increased their hydrodynamic diameter and made them more stable in other biological media (e.g., FBS-containing cell culture media). The thiol-PEG-HS linker (5 kDa) was used on the AuNP surface and to prevent aggregation. In an aqueous suspension with mono-thiol-PEG ligands, AuNPs had long-duration stability in a salt solution buffer and against denaturing agents [29]. Furthermore, the PEGylation of AuNPs inhibits nonspecific adsorption and other interactions, such as opsonization [30,31], avoids the binding of serum proteins, and extends the circulation of a half-life, so that the nanoparticles can accumulate at the tumor site [32].

TEM experiments showed the structural size, size distribution, and shape information on bare and conjugated AuNPs. DLS showed the hydrodynamic diameter, *d_h_*, of the nanoparticles and the presence of nanoparticle aggregates [33], as well as the polydispersity index of the PEGylated bare AuNPs and AuNP-NmAb. The shifted wavelength of the conjugates’ hydrodynamic diameter and the reduced polydispersity index revealed a monolayer of NmAb attached to the AuNPs, similar to the resultsof previous studies [34]. The modification of the PEGylated bare AuNPs with thiol-PEG-NHS reduced the zeta potential. Our results show that PEGylated bare AuNPs had a more negative zeta potential value compared to AuNP-NmAb. If the zeta potential is close to the isoelectric point, the physical stability usually diminishes. Additionally, thiol-PEG-NHS linkers have a large exclusion volume due to their wide hydration layer, which is known to reduce surface contacts between nanoparticles and prevent aggregation [35,36,37,38].

Nanoparticle tracking analysis (NTA) was used to examine bioconjugation, e.g., the attachment of antibodies onto AuNPs. NTA was used to examine how NmAb adhered to gold nanoparticles. Since it has the resolution to discriminate between various populations of similar-sized particles, it was able to show size changes before and after conjugation. The NTA method uses Brownian motion and light scattering to determine the variation in the particle size of samples suspended in a liquid. Every frame of the particle’s movement is recorded. Each detected particle’s center is concurrently located and tracked, and the average distance travelled by every single particle is calculated in the x- and y-axes. The conditions for full conjugation could be determined because the conjugation led to a quantifiable increase in the hydrodynamic radius, which was proportional to the NmAb concentration. The NTA results obtained in this work were validated by comparison to DLS [39].

The stability analyses of the AuNP-NmAb conjugates were performed using 1% NaCl and 10% FBS-containing cell culture medium with an incubation time of 24 h via plasmon resonance by UV-Vis spectroscopy, hydrodynamic diameter, polydispersity index, and zeta-potentials by DLS [22,23,40]. We found that bare AuNPs tend to aggregate in these solutions unpredictably. On the other hand, AuNP-NmAb demonstrated a good colloidal stability [20].

FT-IR measurements further revealed the presence of NmAb on the AuNP surface. In addition, the NH_3_^+^ position changed because of amino acids binding to metal surfaces with a high electron density. The presence of –COO band, –NH and –OH stretching, and NH3+ peak due to amino acid residues are further evidence of NmAb binding onto the nanoparticles. Additionally, FT-IR showed the deformation of the S–H band due to the presence of small quantities of free sulfur in the thiol-PEG-NHS linker in the AuNP-NmAb suspension [41,42].

We further confirmed the conjugation of NmAb onto the AuNP surface by SDS-PAGE analysis under reducing conditions with DTT and elevated heat. While the bare AuNPs that were capped with citrate, tannate, and salt-containing PEG were aggregated and destabilized instantly upon mixing with the loading buffer, AuNP-NmAb did not aggregate, indicating that it is stable. The presence of NmAb was identified by Coomassie blue staining [34]. SDS with DTT-treated free NmAb showed light- and heavy-chain fragments with 20–30 and 40–60 kDa distinctive bands, respectively. The heavy chain of the antibody was covalently bound to AuNPs and, hence, it will not produce fragments easily in a gel [43]. In general, the SDS-PAGE gel results prove the existence of NmAb on the surface of the AuNPs and confirm the functionalization of AuNPs [44].

NmAb efficiently suppresses the proliferation of EGFR+ cancer cells by blocking the EGFR signaling cascade [45]. The effect of NmAb on EGFR+-expressing tumors largely depends on the number of EGFRs on the cell surface [6,46], which can be detected in MTT assays via observing formazan crystal (blue) formation [47]. We evaluated the anti-tumor activity of AuNP-NmAb by measuring the viability of cancer cells (A431, EGFR^medium^, and A549, EGFR^low^) with AuNP-NmAb or free NmAb using the MTT colorimetric assay. Our results are in line with the literature, where radio-labelled NmAb demonstrated a differential efficacy in A431 and A549 cells [48]. In addition, one of our control compounds, cisplatin, showed a highly similar outcome to the previously reported findings [49,50]. Although cisplatin is a capable chemotherapeutic drug, its usage is restricted due to serious systemic side effects and inadequate bioavailability [51]. Our AuNP-NmAb would be better tolerated compared to cisplatin. Moreover, considering nanoparticle toxicity concerns [52], the PEG-AuNP developed in this study could provide a safe approach for future development due to its excellent cytocompatibility. Therefore, AuNP-NmAb could be used as it shows enhanced anti-tumor activity in low- and medium-EGFR-expressing cancer cells compared to NmAb only.

To assess the active targeting capacity and internalization of AuNP-NmAb in A431 (EGFR ^medium^) and A549 (EGFR^low^) cells, AuNP-NmAb or bare AuNPs were added to the cells, and cellular uptake was quantified using spICP-MS, as defined in the Experimental Section. Our results are in agreement with the AuNP conjugates of other mAbs, which also promoted cellular internalization [53,54]. AuNP-NmAb enters the cells via both receptor-mediated endocytosis and transcytosis mechanisms [46]. It was observed that negatively charged or neutral AuNPs migrated and were internalized to a lower degree than the positively charged AuNPs [55]. The internalization of AuNP-NmAb by A431 and A549 cells depend on different levels of EGFR expression. A comprehensive study is needed to identify the underlying process of endocytosis for AuNP-NmAb.

## 5. Conclusions

To our knowledge, this is the first study to demonstrate the successful functionalization of AuNPs’ surface with NmAb, a humanized anti-EGFR IgG1 mAb that targets EGFR and is used against various EGFR-overexpressing cancers. The NmAb was conjugated using an HS-PEG-NHS linker through a coupling reaction. The synthesized spherical 27 nm AuNPs were found to be more stable after PEGylated functionalization. In the presence of NmAb, AuNPs can cause direct cytotoxicity in cancer cells, although NmAb on it is own or bare AuNPs do not have the same anti-cancer effect, even at very high NmAb or AuNP concentrations. The cytotoxicity and cellular uptake of NmAb-functionalized AuNPs are more efficient in skin cancer A431 cells (EGFR^medium^) compared to lung cancer A549 cells (EGFR^low^), very likely due to the different amounts of EGFR expression and cellular uptake. In comparison to NmAb monotherapy, AuNP-NmAb therapy drastically reduced the cell survivability, and the calculated IC_50_ values of AuNP-NmAb were 142.7 and 163.6 µg/mL, while the IC_50_ values of NmAb were 561.3 and 1082.0 µg/mL for the treated A431 and A549 cells, respectively. Thus, this study highlights the unique therapeutic potential of AuNP-NmAb in the EGFR+ target-specific treatment of cancers and promotes further studies involving clinical applications of AuNP-carrier-based mAb therapeutics for enhanced efficacy of mAbs against cancers.

## Figures and Tables

**Table 1:** Comparison of the physicochemical properties of PEGylated bare and conjugated AuNP-NmAb complexes.

Preparations	UV-Vis λ_max_ (nm)	DLS			NTA		TEM (nm)
		Z-Average Size (nm)	PDI	ζ-Potentials (mv)	Mean * (nm)	Mode * (nm)	
Bare AuNPs (PEGylated)	531.33	41.77 ± 1.26	0.315	−35.53 ± 1.7	34.3 ± 0.2	34.7 ± 0.2	27.0 ± 3.0
AuNP-NmAb Conjugates	535.17	58.3 ± 0.36	0.264	−0.048 ± 0.10	64.9 ± 2.9	71.4 ± 1.9	27.21 ± 3.08
AuNP-NmAb +10% NaCl	535.7	59.81 ± 1.18	0.277	−10.2 ± 0.0	-	-	-
AuNPs-NmAb +10% FBS	536.17	73.17 ± 1.14	0.262	−8.54 ± 1.33	-	-	-

*n* = 3 (triplicate of all experiments). * Mean is the average value of studies and Mode is the most repetitive value in the data.

## Data Availability

All data presented in this manuscript is stored in The University of Sydney servers and available upon request.
